# Increased Risk of Diabetes in Inflammatory Bowel Disease Patients: A Nationwide Population-Based Study in Korea

**DOI:** 10.3390/jcm8030343

**Published:** 2019-03-11

**Authors:** Eun Ae Kang, Kyungdo Han, Jaeyoung Chun, Hosim Soh, Seona Park, Jong Pil Im, Joo Sung Kim

**Affiliations:** 1 Department of Internal Medicine and Liver Research Institute, Seoul National University College of Medicine, Seoul 03080, Korea; cheerea@gmail.com (E.A.K.); hosimsoh@gmail.com (H.S.); seven0526@naver.com (S.P.); jp-im@hanmail.net (J.P.I.); jooskim@snu.ac.kr (J.S.K.); 2 Department of Medical Statistics, the Catholic University of Korea College of Medicine, Seoul 06591, Korea; hkd917@naver.com; 3 Department of Internal Medicine, Gangnam Severance Hospital, Yonsei University College of Medicine, Seoul 06229, Korea

**Keywords:** diabetes, epidemiology, inflammatory bowel disease

## Abstract

The association of diabetes with inflammatory bowel disease (IBD) remains unclear. The risk of diabetes in patients with IBD compared with non-IBD controls was investigated. Using the National Health Insurance database of South Korea, 8070 patients with IBD based on the International Classification of Disease 10th revision (ICD-10) codes and rare intractable disease codes for Crohn’s disease (CD) and ulcerative colitis (UC) were compared with 40,350 non-IBD individuals (2010–2014). Newly diagnosed diabetes identified using ICD-10 codes and the prescription of anti-diabetic medication by the end of the follow-up period (2016) was investigated. During a mean follow-up of 5.1 years, the incidence of diabetes in patients with IBD was significantly higher compared with controls after adjusting for serum glucose levels and steroid use (23.19 vs. 22.02 per 1000 person-years; hazard ratio (HR), 1.135; 95% confidence interval (CI), 1.048–1.228). The risk of diabetes was significantly higher in patients with CD (HR, 1.677; 95% CI, 1.408–1.997), but not in UC (HR, 1.061; 95% CI, 0.973–1.156). The effect of IBD on the development of diabetes was significantly more prominent in younger patients (*p* < 0.001). Patients with CD are at a higher risk of diabetes. Regular monitoring for diabetes is recommended, even in younger CD patients who do not use steroid medication.

## 1. Introduction

Diabetes is a metabolic disorder characterized by hyperglycemia due to insulin deficiency or resistance, which results in impaired glucose utilization and eventual multi-organ damage, affecting the eyes, peripheral nerves, cardiovascular and neurovascular structures, and kidneys. Approximately 451 million people worldwide are affected by diabetes and the prevalence is expected to reach 693 million by 2045 [1]. The global increase in diabetes prevalence has been a major threat to healthcare expenditure and public health. In 2017, the global healthcare costs for patients with diabetes were estimated at 850 billion USD, and an estimated 5 million deaths were attributed to diabetes globally [1]. 

Inflammatory bowel disease (IBD), including Crohn’s disease (CD) and ulcerative colitis (UC), is characterized by chronic relapsing inflammation in the gastrointestinal tract related to complex interactions among genetic, environmental, gut microbiome, and immunologic factors [2]. Extraintestinal manifestations from nearly every organ system are developed in 25–40% of patients with IBD [3]. Endocrine and metabolic manifestations in IBD include metabolic bone disease, growth retardation, hypogonadism, pubertal delay, lipid abnormalities, and insulin resistance [4]. However, insulin resistance and hyperglycemia may be a consequence of steroid use in patients with IBD because the steroid induces elevated serum glucose levels via upregulation of hepatic gluconeogenesis, inhibition of glucose uptake in adipose tissue, and impairment of insulin action [5]. Moreover, there is no epidemiological evidence to support that IBD is a definite risk factor for diabetes. In the present study, the incidence and risk factors for diabetes in patients with IBD were determined using a nationwide population-based study and compared with subjects in the general population. 

## 2. Materials and Methods

### 2.1. Data Sources

The National Health Insurance (NHI) service is a mandatory, single public health insurance system that covers most of the Korean population except for 3% of the population covered by Medical Aid due to low income. The NHI database includes demographic information, disease diagnosis, medical procedures, treatments, basic laboratory results, and lifestyle [6]. To reduce the burden of medical costs, the rare and intractable disease (RID) registration system has been established in South Korea for patients with a disease, such as CD, defined as less than 20000 cases. As the number of UC patients is currently increasing to more than 20000, UC is considered an intractable disease. Special diagnostic codes beginning with the letter V were used to identify RIDs. The RID code is reliable and meets the diagnostic criteria set by the NHI, and can only be registered by a physician in South Korea [7]. 

### 2.2. Study Population

A retrospective nationwide cohort study was performed using the NHI database. The National Health Screening Cohort based on NHI data from January 2010 to December 2014 was created, and patients over 20 years of age with IBD were included. Among them, patients with a history of diabetes from 2002 to the time of diagnosis of IBD (index date) were excluded. Patients with IBD were identified using International Classification of Disease 10th revision (ICD-10) and RID registration system codes (V code). The ICD-10 codes for CD and UC are K50 and K51, respectively. The RID codes for CD and UC are V130 and V131, respectively. The patients with IBD met the diagnostic criteria of clinical symptoms, endoscopy, histology, and radiology findings to register both ICD-10 and V codes for CD or UC. The definition of IBD diagnosis based on both ICD-10 and V codes was confirmed using data from a total of 830 patients with IBD at Seoul National University Hospital, a tertiary referral hospital in South Korea. The sensitivities for the diagnosis of CD and UC were 94.5% (312/330) and 96.4% (482/500), respectively [8]. Patients newly diagnosed with IBD between 2010 and 2014 were defined as the incident group. Patients diagnosed with IBD before 2010 and were diagnosed using the same ICD-10 and V codes between 2010 and 2014 were defined as the prevalent group. We identified the history of IBD from 2002 to the index date. The IBD cohort included both incident and prevalent groups. 

### 2.3. Data Collection 

Data were randomly extracted from the general population without a history of CD, UC, or diabetes and matched by age, sex, body mass index (BMI), waist circumference, smoking, alcohol drinking, exercise, and income at a 1:5 ratio on the same index date. The matched control group was considered the non-IBD cohort. Subjects in both IBD and non-IBD cohorts were followed up from the index date to December 2016. Corticosteroid use within the first year from the diagnosis of IBD was analyzed and adjusted. Baseline characteristics including age, sex, BMI, blood pressure, history of smoking and drinking behavior, place of residence, exercise, and initial laboratory findings, including hemoglobin, serum glucose, total cholesterol, alanine aminotransferase (ALT), aspartate aminotransferase (AST), gamma glutamyltransferase (GGT), and triglycerides, were investigated. Smoking behavior was classified as current smoker vs. ex-smoker or non-smoker. Alcohol drinking behavior was defined as ‘yes’ if exceeding 30 g per day. Exercise was defined as moderate-intensity physical activity for 30 min per day, or vigorous-intensity exercise for 20 min per day, at least once a week, based on a questionnaire of the National Health Screening Program [9,10]. Normal waist circumference was defined as less than 90 cm in men and 80 cm in women. Overweight was defined as a BMI over 25. Metabolic syndrome was defined as a combination of hyperglycemia, central obesity, atherogenic dyslipidemia, and hypertension according to the National Cholesterol Education Program Expert Panel and Adult Treatment Panel III criteria. This study was approved by the Institutional Review Board (IRB) of the Seoul National University Hospital (IRB Number, H-1703-107-840).

### 2.4. End Points

The end point of this study was newly diagnosed diabetes during the follow-up period in the IBD and non-IBD cohorts. Diabetes is identified using ICD-10 codes (E11–14) and the prescription of anti-diabetic medication from the NHI database. The operational definition of diabetes based on both ICD-10 codes and prescription data was validated by a previous study [11]. Patients diagnosed with diabetes within the first year from the index date (lag period) were excluded to eliminate the presence of prevalent diabetes which was not causally related to IBD in this study. The comparative risk of newly diagnosed diabetes in the IBD cohort was analyzed based on age, sex, BMI, comorbidities, and baseline glycemic profiles. During the follow-up, patients without newly developed diabetes were censored on the last day of the follow-up or death date. A total of 365 mortality cases (0.75%) were censored in this study. 

### 2.5. Statistical Analysis

Baseline characteristics of IBD and non-IBD patients were analyzed using the Student’s *t*-test for continuous variables with normality assumption and χ^2^ test for categorical variables. A propensity score matching was performed to select non-IBD controls. The incidence rates of diabetes were calculated as the number of incident cases divided by the total follow-up period and presented as per 1000 person-years. The hazard ratio (HR) for the risk of diabetes based on the presence of IBD was calculated after adjusting for covariates such as age, sex, BMI, exercise, smoking, drinking status, BMI, baseline blood glucose level, and steroid use using Cox-proportional hazard models. The incidence probability of diabetes among each group was estimated using Kaplan–Meier analysis and the log-rank test. A *p*-value less than 0.05 was considered statistically significant. SAS version 9.3 (SAS Institute, Cary, NC, USA) and SPSS Version 20.0 (SPSS Inc., Chicago, IL, USA) were used for statistical analyses. 

## 3. Results

### 3.1. Baseline Characteristics 

A total of 8070 IBD patients were compared with 40,350 non-IBD subjects from the general population matched by age, sex, BMI, waist circumference, smoking, drinking, exercise, and income. Among the IBD patients, 1735 patients (21.5%) had CD and 6335 patients (78.5%) had UC. The mean age of the study population was 45.0 ± 12.9 years and mean follow-up duration was 5.1 years. The study population consisted of 32,251 men (66.6%), and the mean age was 45 years. The baseline characteristics of IBD patients and non-IBD controls are shown in Table 1. The non-IBD controls were more likely to live in a rural area (*p* = 0.001) and have more frequent hypertension and dyslipidemia, higher hemoglobin, total cholesterol, serum ALT (alanine aminotransferase), AST (aspartate aminotransferase), GGT (gamma glutamyltransferase), glucose, and triglyceride levels (*p* < 0.001). The non-IBD controls used less steroids (*p* < 0.001). The baseline characteristics of both CD and UC patients compared with non-IBD subjects are shown in Supplementary Table S1.

### 3.2. Incidence and Risk of Diabetes

During the follow-up, the incidence rate of diabetes in IBD patients and non-IBD controls was 23.19 and 22.02 per 1000 person-years, respectively (Table 2). The risk of diabetes in patients with IBD was significantly higher than in non-IBD controls after adjusting for age, sex, smoking, drinking, and exercise (hazard ratio (HR), 1.091; 95% confidence interval (CI), 1.008–1.180, *p* = 0.031). The adjusted HR for diabetes was 1.410 (95% CI, 1.185–1.679; *p* < 0.001) in CD and 1.040 (95% CI, 0.955–1.133) in UC, respectively, compared with non-IBD controls (Model 3 in Table 2). The adjusted HRs for diabetes when adjusting for BMI and baseline blood glucose level also demonstrated a significantly higher risk of diabetes in the IBD cohort compared with the non-IBD cohort (HR, 1.154; 95% CI, 1.067–1.249; *p* < 0.001; Model 4 in Table 2). In addition, after adjusting for steroid use, the risk of diabetes in the IBD cohort was significantly higher than in the non-IBD cohort (HR, 1.135; 95% CI, 1.048–1.228; *p* = 0.002; Model 5 in Table 2). The HR for diabetes was 1.677 (95% CI, 1.408–1.997; *p* < 0.001) compared with the non-IBD cohort even after adjusting for age, sex, smoking, drinking, exercise, BMI, baseline blood glucose level, and steroid use, especially in CD patients. However, the risk of diabetes between non-IBD and UC cohorts after adjusting for age, sex, smoking, drinking, exercise, BMI, baseline blood glucose level, and steroid use was not significantly different (adjusted HR, 1.061; 95% CI, 0.973–1.156).

### 3.3. Subgroup Analysis

**Figure 1:**
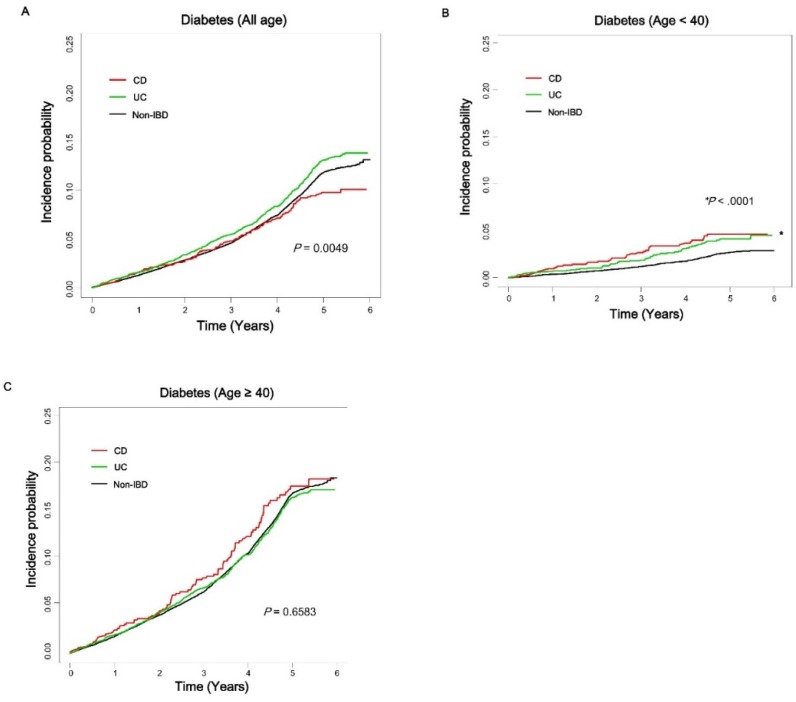
Incidence probability of diabetes in patients with Crohn’s disease (CD) and ulcerative colitis (UC) compared with age-matched non-IBD (inflammatory bowel disease) controls based on age group. Patients with CD (red line), UC (green line), and non-IBD controls (black line). All ages (**A**), under 40 years (**B**), over 40 years (**C**). CD, Crohn’s disease; IBD, inflammatory bowel disease; UC, ulcerative colitis.

**Figure 2:**
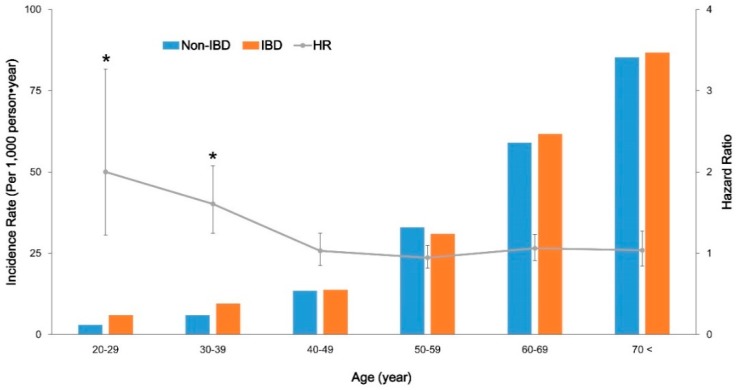
Incidence rate (left *Y* axis, column) and hazard ratio (right *Y* axis, line with 95% confidence intervals; CI) of diabetes in patients with inflammatory bowel disease (IBD) compared with non-IBD controls based on age group. Error bars represent 95% CIs. * Indicates *p* < 0.05; HR, hazard ratio; IBD, inflammatory bowel disease; UC, ulcerative colitis.

The age-related incidence rate of diabetes in patients with IBD compared with non-IBD controls was analyzed (Figure 1). The incidence probabilities among CD, UC, and non-IBD cohorts, including all ages of the study population, were not significantly different (Figure 1A). However, patients with CD and UC showed significantly higher incidence probabilities compared with non-IBD subjects in the under 40 years of age subgroup (Figure 1B). The difference in incidence probabilities between IBD and non-IBD cohorts was less significant in the over 40 years of age subgroup (Figure 1C). The incidence rate of diabetes (per 1000 person-years) in IBD patients and controls in their 20s was 6.0 and 3.0, respectively (HR, 2.001; 95% CI 1.226–3.265; *p* = 0.0055). In their 30s, the incidence rate of diabetes (per 1000 person–years) in IBD patients and controls was 9.5 and 6.0, respectively (HR, 1.608; 95% CI 1.245–2.077; *p* = 0.0003). However, in individuals over 40 years of age, the difference of incidence rates of diabetes between IBD and non-IBD cohorts was not significant (Supplementary Table S2 and Figure 2). The incidence rate of diabetes increased based on age in both cohorts; however, the HR of diabetes was significantly higher in younger IBD patients compared with same-aged non-IBD controls. The effect of both CD and UC on the development of diabetes was significantly more prominent in younger patients under 40 years of age (in CD: HR, 2.395 vs. 1.563; in UC: HR, 1.589 vs. 1.020; *p*-value based on interaction analysis, 0.0026). The effect of IBD on the development of diabetes among subgroups classified based on sex, waist circumference, metabolic syndrome, overweight, and steroid use was not significantly different (Table 3).

## 4. Discussion

In the present Korean nationwide population-based study, the relationship between IBD and diabetes was investigated. After analyzing a total of 48,420 individuals in the NHI database, the incidence of diabetes in patients with IBD, and especially with CD, was significantly higher compared with subjects in the general population matched by age, sex, BMI, smoking, alcohol drinking, exercise, and income. To the best of our knowledge, this is the first epidemiologic study in which the incidence and risk of diabetes in patients with IBD have been evaluated.

Based on a literature review, the causal relationship between IBD and diabetes is still controversial. In a cross-sectional study including 12,601 patients with IBD, type-1 diabetes was the third most common comorbidity in IBD patients (prevalence, 1.0%); however, the odds for type-1 diabetes were not significantly elevated in both CD and UC patients [12]. A cross-sectional study using claims data in the United States reported that there was no significant association between IBD and type-1 diabetes [13]. A recent epidemiologic study of the NHI Survey in the United States also showed that the age-adjusted prevalence of type-1 and -2 diabetes was 10.1 (95% CI, 8.2–12.4) in patients with IBD and 8.6 (95% CI, 8.4–8.9) in individuals without IBD, respectively, but the difference was not statistically significant [14]. In contrast, a recent cross-sectional study from 47,325 IBD patients in Denmark showed that type-1 diabetes was significantly associated with both UC and CD [15]. In a case-control study of 1200 pediatric IBD patients, the prevalence of type-1 and -2 diabetes was also higher in UC patients than in controls (odds ratio, 2.7; 95% CI, 1.1–6.6) [16]. The first population-based cohort study from the United Kingdom Clinical Practice Research Datalink demonstrated a crude IBD incidence rate of 37.7 per 100,000 person-years in 141,170 patients with type-2 diabetes, and the use of dipeptidyl peptidase-4 inhibitors was associated with an increased risk of UC, but not CD [17]. In the present study, the risk of type-1 and -2 diabetes after adjusting for steroid use and all risk factors for diabetes including age, sex, smoking, drinking, exercise, and BMI, the baseline blood glucose level was significantly higher in patients with CD, but not in patients with UC. Considering the results of large-scale population-based cohort studies, there may be a potential risk of type-1 and -2 diabetes in IBD, although there is still debate surrounding the causal relationship between IBD and diabetes due to the heterogeneity of the study design and population.

The mechanism of chronic inflammation and genetic factors common to type-1 and -2 diabetes and IBD, especially CD, may play a crucial role in this phenomenon. One explanation for the relationship between IBD and diabetes is that IBD is associated with diabetes in terms of chronic inflammation and dysbiosis [18,19]. Increased permeability of the intestinal mucosa and disturbance of gut microbiota can activate systemic inflammation through T helper 17 lymphocytes, eventually leading to autoimmune diseases such as IBD and type-1 diabetes [20,21]. Crosstalk between diabetes and IBD is possible, due to the mechanisms involved in the breakdown of homeostasis [20]. Jurjus A et al. suggested that IBD and type-2 diabetes were linked through dysbiosis and multiple inflammatory consequences [18]. In several studies, adiponectin, leptin, and ghrelin levels in IBD patients differed from individuals without IBD [22,23,24]. Hypothetically, the correlation between IBD and diabetes diseases is based on common multiple inflammatory pathways such as nuclear factor kappa-B (NF-κB) [25]. However, the relationship between diabetes and IBD remains unclear and conflicting [26]. In previous studies on pathophysiology and genetic mutations of IBD and diabetes, evidence of genetic or epigenetic predisposition, common to both IBD and diabetes, was found. Several genetic loci variants related to the pathogenesis of IBD and diabetes have been reported. The gene mutations in protein tyrosine phosphatase non-receptor type (PTPN)2 and PTPN22 are a genetic variation that plays a role in the development of both CD and type-1 diabetes [27]. In type-1 diabetes, the PTPN2 mutation plays an important role in apoptosis of pancreatic beta cells but modulates intestinal epithelial barrier function and the innate immune response in CD [28,29,30,31]. However, in another study, the risk alleles for type-1 diabetes such as PTPN22, interleukin (IL)-27, and IL-10 loci were shown to protect against CD [32]. Regarding gut microbiome and dysbiosis, bile acid modification genes in gut microbiome were abundant in CD and type-2 diabetes patients [33].

The overall incidence of diabetes increases with age; however, IBD patients may be at a significant risk of developing diabetes even at a younger age compared with the general population. In the subgroup analysis in this study, a more prominent effect of CD and UC was found on the development of diabetes in younger age groups, especially those under 40 years of age. Younger CD patients under 40 years of age had a 2.4-fold higher risk of diabetes compared with the non-IBD controls. Even patients under 40 years of age with UC had a significantly higher risk of diabetes compared with the general population. These findings indicate that IBD has a critical role in the pathogenesis of diabetes in younger individuals at a relatively low risk of metabolic diseases. Therefore, physicians should be aware that young patients with IBD could develop diabetes. Shanahan F et al. reported that microbial disturbances, as an environmental factor in early life, contribute to the development of chronic inflammation and metabolic disorders [34]. Because insulin resistance is also associated with chronic inflammation, IBD can increase the risk of diabetes through inflammatory and metabolic signaling in younger patients [35]. Early exposure to environmental factors which affect the proportion of microbiota, such as early antibiotic exposure, increases the risk of IBD [36,37]. These environmental and genetic factors in young patients with IBD may affect glucose metabolism and result in diabetes. However, the pathogenesis of diabetes in younger patients remains unclear and further investigations are warranted.

This study had several limitations including the retrospective cohort design. Type-1 and -2 diabetes, as study end points, could not be distinguished in this study population. In addition, the risk of diabetes based on the severity of IBD could not be demonstrated because the NHI database had no information regarding the severity of IBD. However, the risk of developing diabetes related to IBD, especially CD in younger patients, was also significant regardless of steroid use, indicating the severe disease course of IBD. Further prospective studies may be needed to determine the risk of diabetes based on the severity of IBD. The study design, i.e., comparing IBD and non-IBD cohorts matched by multiple variables, might reveal intermediary factors in a possible causal relationship between IBD and diabetes due to overmatching.

## 5. Conclusions

In conclusion, patients with CD have a significantly increased risk of diabetes compared with non-IBD controls, regardless of steroid use. Therefore, regular monitoring of blood glucose levels should be considered for the early detection of diabetes in patients with CD, even in younger patients.

## Figures and Tables

**Table 1:** Baseline characteristics of the study population.

	IBD	Non-IBD	*p*-Value
No. of patients	8070	40,350	
Age, years *	45.0 ± 12.8	44.9 ± 13.1	0.3230
Male^†^ (%)	5371 (66.6)	26,880 (66.6)	0.9142
Height, cm *	166.4 ± 8.8	166.4 ± 8.6	0.5713
Body weight, kg *	63.4 ± 11.2	63.3 ± 11.0	0.4491
BMI, kg/m^2^ *	22.8 ± 3.0	22.8 ± 2.9	0.6091
<18.5 ^†^	518 (6.4)	2549 (6.3)	0.6280
18.5–25.0 ^†^	5762 (71.4)	29,023 (71.9)	
>25.0 ^†^	1790 (22.2)	8778 (21.8)	
Waist circumference, cm *	78.8 ± 8.7	78.6 ± 8.7	0.1619
Residence; Urban ^†^ (%)	3902 (48.4)	18,677 (46.3)	0.0008
Smoking; Current ^†^ (%)	1133 (14.0)	5961 (14.8)	0.0889
Drinking; No ^†^ (%)	7809 (96.8)	38,997 (96.7)	0.5868
Exercise; Yes ^†^ (%)	4705 (58.3)	23,480 (58.2)	0.8529
Underlying illness			
Hypertension ^†^	1295 (16.1)	7376 (18.3)	<0.0001
Systolic BP, mmHg *	118.5 ± 13.7	120.2 ± 14.1	<0.0001
Diastolic BP, mmHg *	74.1 ± 9.4	75.1 ± 9.7	<0.0001
Dyslipidemia ^†^	912 (11.3)	5896 (14.6)	<0.0001
Corticosteroid use; Yes ^†^ (%)	4080 (50.6)	14,810 (36.7)	<0.0001
Initial laboratory findings *		
Glucose, mg/dL	91.4 ± 10.9	92.6 ± 11.2	<0.0001
Total cholesterol, mg/dL	182.6 ± 35.6	191.7 ± 35.4	<0.0001
Hemoglobin, g/dL	13.8 ± 1.7	14.2 ± 1.6	<0.0001
ALT, IU/L ^‡^	19.3 (19.1–19.5)	21.5 (21.4–21.6)	<0.0001
AST, IU/L ^‡^	22.8 (22.6–22.9)	23.8 (23.7–23.9)	<0.0001
GGT, IU/L ^‡^	23.1 (22.8–23.4)	25.2 (25.0–25.4)	<0.0001
Triglyceride, mg/d L ^‡^	99.0 (97.9–100.2)	103.8 (103.2–104.4)	<0.0001

ALT, alanine aminotransferase; AST, aspartate aminotransferase; BMI, body mass index; BP, blood pressure; GGT, gamma glutamyltransferase; IBD, inflammatory bowel disease; IU, international unit; * Mean ± standard deviation; ^†^ Number (%); ^‡^ Median (95% confidence interval).

**Table 2:** Incidence and risk of diabetes in inflammatory bowel disease.

	N	Diabetes	IR	HR (95% CI) *				
				Model 1 ^†^	Model 2 ^‡^	Model 3 ^§^	Model 4 ^‖^	Model 5
Control	40,350	3724	22.02	1 (Ref.)	1 (Ref.)	1 (Ref.)	1 (Ref.)	1 (Ref.)
IBD	8070	743	23.19	1.064 (0.984–1.152)	1.088 (1.006–1.177)	1.091 (1.008–1.180)	1.154 (1.067–1.249)	1.135 (1.048–1.228)
*p*–value				0.1209	0.0356	0.0307	0.0004	0.0018
CD	1735	132	18.83	0.859 (0.722–1.021)	1.420 (1.193–1.691)	1.410 (1.185–1.679)	1.697 (1.425–2.020)	1.677 (1.408–1.997)
UC	6335	611	24.41	1.122 (1.030–1.223)	1.036 (0.951–1.129)	1.040 (0.955–1.133)	1.080 (0.991–1.176)	1.061 (0.973–1.156)
*p*–value				0.0049	0.0004	0.0005	<0.0001	<0.0001

CD, Crohn’s disease; CI, confidence intervals; HR, hazard ratios; IBD, inflammatory bowel disease; IR, incidence rates; N, number; Ref., reference; UC, ulcerative colitis; * Median (95% confidence interval); ^†^ Model 1: Non-adjusted; ^‡^ Model 2: Adjusted by age and sex; ^§^ Model 3: Adjusted by age, sex, smoke, drink, and exercise; ^‖^ Model 4: Adjusted by age, sex, smoke, drink, exercise, BMI, and baseline blood glucose level, Model 5: Adjusted by age, sex, smoke, drink, exercise, BMI, baseline blood glucose level, and steroid use.

**Table 3:** Subgroup analysis of risk for diabetes in inflammatory bowel disease.

Subgroup		IBD	HR (95% CI)	*p*-Value *	*p*-Value for Interaction Analysis ^†^
Age	<40	CD	2.395 (1.715–3.345)	<0.0001	0.0026
		UC	1.589 (1.198–2.108)		
	≥40	CD	1.563 (1.27–1.924)	0.0001	
		UC	1.020 (0.932–1.116)		
Sex	Male	CD	1.674 (1.352–2.072)	<0.0001	0.8492
		UC	1.074 (0.965–1.195)		
	Female	CD	1.745 (1.284–2.371)	0.0017	
		UC	1.045 (0.903–1.201)		
Waist Circumference	No	CD	1.661 (1.352–2.040)	<0.0001	0.7684
		UC	1.075 (0.967–1.195)		
	Yes	CD	1.714 (1.229–2.391)	0.0063	
		UC	1.036 (0.892–1.202)		
Metabolic syndrome	No	CD	1.832 (1.480–2.269)	<0.0001	0.3152
		UC	1.147 (1.020–1.290)		
	Yes	CD	1.576 (1.160–2.141)	0.0120	
		UC	1.052 (0.926–1.195)		
Overweight	No	CD	1.682 (1.364–2.073)	<0.0001	0.4154
		UC	1.006 (0.902–1.122)		
	Yes	CD	1.656 (1.203–2.28)	0.0011	
		UC	1.172 (1.018–1.348)		
Steroid use	No	CD	1.558 (1.204–2.016)	0.0027	0.4088
		UC	1.060 (0.932–1.206)		
	Yes	CD	1.781 (1.403–2.260)	<0.0001	
		UC	1.051 (0.936–1.180)		

IBD, inflammatory bowel disease; HR, hazard ratios; CI, confidence intervals; CD, Crohn’s disease; UC, ulcerative colitis; * *p*-value represents the statistical significance of HR of diabetes in each subgroup; ^†^ A *p*-value < 0.05 for interaction analysis implies statistically significant subgroup interactions with HR of diabetes in IBD patients.

## Supplementary Materials

The following are available online at https://www.mdpi.com/2077-0383/8/3/343/s1, Table S1: Baseline characteristics of CD and UC patients compared to non-IBD cohort, Table S2: Incidence rate of diabetes according to age group between IBD and non-IBD cohort.

## List of Abbreviations

ALT, alanine aminotransferase; AST, aspartate aminotransferase; BMI, body mass index; CD, Crohn’s disease; CI, confidence interval; GGT, gamma glutamyltransferase; HR, hazard ratio; IBD, inflammatory bowel disease; ICD, International Classification of Disease; IL, interleukin; NF-κB, nuclear factor-kappa B; NHI, National Health Insurance; PTPN, protein tyrosine phosphatase, non-receptor type; RID, rare and intractable disease; UC, ulcerative colitis.

## References

[1] Cho N.H., Shaw J.E., Karuranga S., Huang Y., Fernandes J.D.D., Ohlrogge A.W., Malanda B. (2018). IDF Diabetes Atlas: Global estimates of diabetes prevalence for 2017 and projections for 2045. Diabetes Res. Clin. Pract..

[2] Basso P.J., Fonseca M.T., Bonfa G., Alves V.B., Sales-Campos H., Nardini V., Cardoso C.R. (2014). Association among genetic predisposition, gut microbiota, and host immune response in the etiopathogenesis of inflammatory bowel disease. Braz. J. Med. Biol. Res..

[3] Bernstein C.N., Blanchard J.F., Rawsthorne P., Yu N. (2001). The prevalence of extraintestinal diseases in inflammatory bowel disease: A population–based study. Am. J. Gastroenterol..

[4] Tigas S., Tsatsoulis A. (2012). Endocrine and metabolic manifestations in inflammatory bowel disease. Ann. Gastroenterol..

[5] van Raalte D.H., Ouwens D.M., Diamant M. (2009). Novel insights into glucocorticoid–mediated diabetogenic effects: Towards expansion of therapeutic options?. Eur. J. Clin. Investig..

[6] Song S.O., Jung C.H., Song Y.D., Park C.Y., Kwon H.S., Cha B.S., Park J.Y., Lee K.U., Ko K.S., Lee B.W. (2014). Background and data configuration process of a nationwide population–based study using the korean national health insurance system. Diabetes Metab. J..

[7] Kim H.J., Hann H.J., Hong S.N., Kim K.H., Ahn I.M., Song J.Y., Lee S.H., Ahn H.S. (2015). Incidence and natural course of inflammatory bowel disease in Korea, 2006–2012: A nationwide population–based study. Inflamm. Bowel Dis..

[8] Park S., Chun J., Han K.D., Soh H., Choi K., Kim J.H., Lee J., Lee C., Im J.P., Kim J.S. (2018). Increased end–stage renal disease risk in patients with inflammatory bowel disease: A nationwide population–based study. World J. Gastroenterol..

[9] Task Force on Community Preventive Services (2009). A recommendation to improve employee weight status through worksite health promotion programs targeting nutrition, physical activity, or both. Am. J. Prev. Med..

[10] Choi K.M., Han K., Park S., Chung H.S., Kim N.H., Yoo H.J., Seo J.A., Kim S.G., Kim N.H., Baik S.H. (2018). Implication of liver enzymes on incident cardiovascular diseases and mortality: A nationwide population-based cohort study. Sci. Rep..

[11] Lee Y.H., Han K., Ko S.H., Ko K.S., Lee K.U. (2016). Data Analytic Process of a Nationwide Population-Based Study Using National Health Information Database Established by National Health Insurance Service. Diabetes Metab. J..

[12] Weng X., Liu L., Barcellos L.F., Allison J.E., Herrinton L.J. (2007). Clustering of inflammatory bowel disease with immune mediated diseases among members of a northern california-managed care organization. Am. J. Gastroenterol..

[13] Cohen R., Robinson D., Paramore C., Fraeman K., Renahan K., Bala M. (2008). Autoimmune disease concomitance among inflammatory bowel disease patients in the United States, 2001–2002. Inflamm. Bowel Dis..

[14] Xu F., Dahlhamer J.M., Zammitti E.P., Wheaton A.G., Croft J.B. (2018). Health-Risk Behaviors and Chronic Conditions Among Adults with Inflammatory Bowel Disease—United States, 2015 and 2016. MMWR Morb. Mortal. Wkly. Rep..

[15] Halling M.L., Kjeldsen J., Knudsen T., Nielsen J., Hansen L.K. (2017). Patients with inflammatory bowel disease have increased risk of autoimmune and inflammatory diseases. World J. Gastroenterol..

[16] Kappelman M.D., Galanko J.A., Porter C.Q., Sandler R.S. (2011). Association of paediatric inflammatory bowel disease with other immune-mediated diseases. Arch. Dis. Child..

[17] Abrahami D., Douros A., Yin H., Yu O.H.Y., Renoux C., Bitton A., Azoulay L. (2018). Dipeptidyl peptidase-4 inhibitors and incidence of inflammatory bowel disease among patients with type 2 diabetes: Population based cohort study. BMJ.

[18] Jurjus A., Eid A., Al Kattar S., Zeenny M.N., Gerges-Geagea A., Haydar H., Hilal A., Oueidat D., Matar M., Tawilah J. (2016). Inflammatory bowel disease, colorectal cancer and type 2 diabetes mellitus: The links. BBA Clin..

[19] Kamada N., Seo S.U., Chen G.Y., Nunez G. (2013). Role of the gut microbiota in immunity and inflammatory disease. Nat. Rev. Immunol..

[20] Morris G., Berk M., Carvalho A.F., Caso J.R., Sanz Y., Maes M. (2016). The Role of Microbiota and Intestinal Permeability in the Pathophysiology of Autoimmune and Neuroimmune Processes with an Emphasis on Inflammatory Bowel Disease Type 1 Diabetes and Chronic Fatigue Syndrome. Curr. Pharm. Des..

[21] Arif S., Moore F., Marks K., Bouckenooghe T., Dayan C.M., Planas R., Vives-Pi M., Powrie J., Tree T., Marchetti P. (2011). Peripheral and islet interleukin-17 pathway activation characterizes human autoimmune diabetes and promotes cytokine-mediated beta-cell death. Diabetes.

[22] Karmiris K., Koutroubakis I.E., Xidakis C., Polychronaki M., Voudouri T., Kouroumalis E.A. (2006). Circulating levels of leptin, adiponectin, resistin, and ghrelin in inflammatory bowel disease. Inflamm. Bowel Dis..

[23] Trejo-Vazquez F., Garza-Veloz I., Villela-Ramirez G.A., Ortiz-Castro Y., Mauricio-Saucedo P., Cardenas-Vargas E., Diaz-Baez M., Cid-Baez M.A., Castaneda-Miranda R., Ortiz-Rodriguez J.M. (2018). Positive association between leptin serum levels and disease activity on endoscopy in inflammatory bowel disease: A case-control study. Exp. Ther. Med..

[24] Peng Y.J., Shen T.L., Chen Y.S., Mersmann H.J., Liu B.H., Ding S.T. (2018). Adiponectin and adiponectin receptor 1 overexpression enhance inflammatory bowel disease. J. Biomed. Sci..

[25] Andlujar I., Recio M.C., Giner R.M., Cienfuegos-Jovellanos E., Laghi S., Muguerza B., Rios J.L. (2011). Inhibition of Ulcerative Colitis in Mice after Oral Administration of a Polyphenol-Enriched Cocoa Extract Is Mediated by the Inhibition of STAT1 and STAT3 Phosphorylation in Colon Cells. J. Agric. Food Chem..

[26] Bahler C., Schoepfer A.M., Vavricka S.R., Brungger B., Reich O. (2017). Chronic comorbidities associated with inflammatory bowel disease: Prevalence and impact on healthcare costs in Switzerland. Eur. J. Gastroenterol. Hepatol..

[27] Sharp R.C., Abdulrahim M., Naser E.S., Naser S.A. (2015). Genetic Variations of PTPN2 and PTPN22: Role in the Pathogenesis of Type 1 Diabetes and Crohn’s Disease. Front. Cell. Infect. Microbiol..

[28] Santin I., Moore F., Colli M.L., Gurzov E.N., Marselli L., Marchetti P., Eizirik D.L. (2011). PTPN2, a candidate gene for type 1 diabetes, modulates pancreatic beta-cell apoptosis via regulation of the BH3-only protein Bim. Diabetes.

[29] McCole D.F. (2012). Regulation of epithelial barrier function by the inflammatory bowel disease candidate gene, PTPN2. Ann. N. Y. Acad. Sci..

[30] Spalinger M.R., Kasper S., Chassard C., Raselli T., Frey-Wagner I., Gottier C., Lang S., Atrott K., Vavricka S.R., Mair F. (2015). PTPN2 controls differentiation of CD4(+) T cells and limits intestinal inflammation and intestinal dysbiosis. Mucosal Immunol..

[31] Scharl M., Paul G., Weber A., Jung B.C., Docherty M.J., Hausmann M., Rogler G., Barrett K.E., McCole D.F. (2009). Protection of epithelial barrier function by the Crohn’s disease associated gene protein tyrosine phosphatase n2. Gastroenterology.

[32] Wang K., Baldassano R., Zhang H., Qu H.Q., Imielinski M., Kugathasan S., Annese V., Dubinsky M., Rotter J.I., Russell R.K. (2010). Comparative genetic analysis of inflammatory bowel disease and type 1 diabetes implicates multiple loci with opposite effects. Hum. Mol. Genet..

[33] Labbe A., Ganopolsky J.G., Martoni C.J., Prakash S., Jones M.L. (2014). Bacterial bile metabolising gene abundance in Crohn’s, ulcerative colitis and type 2 diabetes metagenomes. PLoS ONE.

[34] Shanahan F., Sheehan D. (2016). Microbial contributions to chronic inflammation and metabolic disease. Curr. Opin. Clin. Nutr. Metab. Care.

[35] Shanahan F. (2012). The gut microbiota-a clinical perspective on lessons learned. Nature reviews. Gastroenterol. Hepatol..

[36] Bernstein C.N., Shanahan F. (2008). Disorders of a modern lifestyle: Reconciling the epidemiology of inflammatory bowel diseases. Gut.

[37] Shaw S.Y., Blanchard J.F., Bernstein C.N. (2010). Association between the use of antibiotics in the first year of life and pediatric inflammatory bowel disease. Am. J. Gastroenterol..

