# Supplementary material for: Increased Risk of Diabetes in Inflammatory Bowel Disease Patients: A Nationwide Population-Based Study in Korea
Source: J Clin Med. 2019 Mar 11;8(3):343. doi: 10.3390/jcm8030343 (PMC6463263; doi:10.3390/jcm8030343)
Supplement: Supplementary file 1 [file jcm-08-00343-s001.pdf]

Supplementary Table S1. Baseline characteristics of CD and UC patients compared to non-IBD cohort

	CD	UC	Non-IBD	*P*-value
No. of patients	1,735	6,335	40,350	
Age, years*	38.5 ± 11.7	46.8 ± 12.5	44.9 ± 13.1	< .0001
Male† (%)	1,300 (74.9)	4,071 (64.3)	26,880 (66.6)	0.0088
Height, cm*	168.6 ± 8.7	165.8 ± 8.7	166.4 ± 8.6	< .0001
Body weight, kg*	62.4 ± 11.3	63.7 ± 11.1	63.3 ± 11.0	< .0001
BMI, kg/m^2*	21.9 ± 3.1	23.1 ± 2.9	22.8 ± 2.9	< .0001
< 18.5†	218 (12.6)	300 (4.7)	2,549 (6.3)	< .0001
18.5 – 25.0†	1,241 (71.5)	4,521 (71.4)	29,023 (71.9)	< .0001
> 25.0†	276 (15.9)	1,514 (23.9)	8,778 (21.8)	< .0001
Waist circumference, cm*	77.0 ± 8.6	79.2 ± 8.6	78.6 ± 8.7	< .0001
Residence; Urban† (%)	855 (49.3)	3,047 (48.1)	18,677 (46.3)	0.0049
Smoking; Current† (%)	385 (22.2)	748 (11.8)	5,961 (14.8)	< .0001
Drinking; No† (%)	1,682 (96.7)	6,127 (96.7)	38,997 (96.7)	0.1133
Exercise; Yes† (%)	1,018 (58.7)	3,687 (58.2)	23,480 (58.2)	< .0001
Underlying illness				
Hypertension†	131 (7.6)	1,164 (18.4)	7,376 (18.3)	< .0001
Systolic BP, mmHg*	115.4 ± 13.2	119.4 ± 13.7	120.2 ± 14.1	< .0001
Diastolic BP, mmHg*	72.2 ± 9.1	74.7 ± 9.4	75.1 ± 9.7	< .0001
Dyslipidemia†	87 (5.0)	825 (13.0)	5,896 (14.6)	< .0001
Corticosteroid use; Yes† (%)	796 (45.9)	3,284 (51.8)	14,810 (36.7)	< .0001
Initial laboratory findings*				
Glucose, mg/dL	89.3 ± 10.5	91.9 ± 10.9	92.6 ± 11.2	< .0001
Total cholesterol, mg/dL	164.2 ± 34.3	187.6 ± 34.3	191.7 ± 35.4	< .0001
Hemoglobin, g/dL	13.4 ± 1.8	13.9 ± 1.7	14.2 ± 1.6	< .0001
ALT, IU/L‡	17.2 (16.8-17.6)	20.0 (19.7-20.2)	21.5 (21.4 - 21.6)	< .0001
AST, IU/L‡	21.2 (21.0-21.5)	23.2 (23.1-23.4)	23.8 (23.7 - 23.9)	< .0001
GGT, IU/L‡	21.7 (21.1-22.3)	23.5 (23.2-23.9)	25.2 (25.0 – 25.4)	< .0001

	CD	UC	Non-IBD	*P*-value
Triglyceride, mg/dL‡	96.5 (94.3-98.8)	99.8 (98.5-101.0)	103.8 (103.2 – 104.4)	< .0001

ALT, alanine aminotransferase; AST, aspartate aminotransferase; BMI, body mass index; BP, blood pressure; GGT, gamma glutamyltransferase; IBD, inflammatory bowel disease; IU, international unit

* Mean ± standard deviation

† Number (%)

‡ Median (95% confidence interval)

Supplementary Table S2. Incidence rate of diabetes according to age group between IBD and non-IBD cohort

Age	IBD	N	Diabetes (n)	Duration (person·year)	IR	HR (95% CI)*	*P*-value
20-29	NO	5,029	59	19725.73	2.991	1	
	YES	938	22	3673.19	5.9893	2.001 (1.226-3.265)	0.0055
30-39	NO	9,970	260	43544.29	5.9709	1	
	YES	1,992	76	8021.03	9.4751	1.608 (1.245-2.077)	0.0003
40-49	NO	10,997	626	46660.31	13.4161	1	
	YES	2,218	124	9012.07	13.7593	1.030 (0.850-1.249)	0.7607
50-59	NO	8,227	1142	34582.59	33.0224	1	
	YES	1,709	211	6812.73	30.9714	0.945 (0.816-1.095)	0.4536
60-69	NO	4,285	1034	17505.2	59.0682	1	
	YES	872	204	3303.65	61.7498	1.061 (0.913-1.233)	0.4398
> 70	NO	1,842	603	7068.67	85.306	1	
	YES	341	106	1222.11	86.7355	1.037 (0.843-1.274)	0.7328

CI, confidence intervals; HR, hazard ratios; IBD, inflammatory bowel disease; IR, incidence rates; N, number

* Median (95% confidence interval)
